# THE RELATIONSHIP BETWEEN CROSS-TISSUE AND INTRA-TISSUE MEASURES OF AGING BIOLOGY IN OLDER ADULTS

**DOI:** 10.1093/geroni/igad104.2774

**Published:** 2023-12-21

**Authors:** Reem Waziry

**Affiliations:** Columbia University, New York City, New York, United States

## Abstract

The present study included individuals (N=2406) aged 50 and older who had complete data for saliva-based telomere length and blood-based biological aging measures in the Health and Retirement Venous blood and Telomere length studies. Average telomere length was assayed using quantitative PCR (qPCR). DNA methylation clocks included: Horvath (353 CpG sites), Hannum (71 CpG sites), Levine (referred to as phenoAge, (513 CpG sites)), GrimAge, (epigenetic surrogate markers for select plasma proteins), Horvath Skin and Blood (391 CpG sites), Lin (99 CpG sites), Weidner (3 CpG sites), and VidalBralo (8 CpG sites). Physiology measures included albumin, creatinine, glucose, [log] C-reactive protein, lymphocyte percent, mean cell volume, red blood cell distribution width, alkaline phosphatase, and white blood cell count. Relationships between aging biology measures in blood and saliva and variations according to gender were assessed. Blood-based measures showed an inverse relationship with saliva-based telomere length. Increase in saliva-based telomere length was associated with 1 to 4 years slower biological aging based on blood-based measures with the highest magnitude being Weidner (Coefficient= -3.97, P = 0.005), GrimAge (-3.33, P < 0.001), and Lin (-3.45, P = 0.008). Variations in DNA methylation and physiology measures of aging biology with changes in telomere length provide new avenues for integration of these measures in clinical care of vulnerable and clinically difficult to reach individuals.

